# Three-Dimensional Ordered Mesoporous Carbon Spheres Modified with Ultrafine Zinc Oxide Nanoparticles for Enhanced Microwave Absorption Properties

**DOI:** 10.1007/s40820-021-00601-x

**Published:** 2021-02-17

**Authors:** Yan Song, Fuxing Yin, Chengwei Zhang, Weibing Guo, Liying Han, Ye Yuan

**Affiliations:** School of Materials Science and Engineering, Tianjin Key Laboratory of Materials Laminating Fabrication and Interface Control Technology, Hebei University of Technology, Tianjin, 300130 People’s Republic of China

**Keywords:** Three-dimensional ordered structure, Mesoporous carbon spheres, Zinc oxide nanoparticles, Microwave absorption

## Abstract

Currently, electromagnetic radiation and interference have a significant effect on the operation of electronic devices and human health systems. Thus, developing excellent microwave absorbers have a huge significance in the material research field. Herein, a kind of ultrafine zinc oxide (ZnO) nanoparticles (NPs) supported on three-dimensional (3D) ordered mesoporous carbon spheres (ZnO/OMCS) is prepared from silica inverse opal by using phenolic resol precursor as carbon source. The prepared lightweight ZnO/OMCS nanocomposites exhibit 3D ordered carbon sphere array and highly dispersed ultrafine ZnO NPs on the mesoporous cell walls of carbon spheres. ZnO/OMCS-30 shows microwave absorbing ability with a strong absorption (− 39.3 dB at 10.4 GHz with a small thickness of 2 mm) and a broad effective absorption bandwidth (9.1 GHz). The outstanding microwave absorbing ability benefits to the well-dispersed ultrafine ZnO NPs and the 3D ordered mesoporous carbon spheres structure. This work opened up a unique way for developing lightweight and high-efficient carbon-based microwave absorbing materials.

**Electronic supplementary material:**

The online version of this article (10.1007/s40820-021-00601-x) contains supplementary material, which is available to authorized users.

## Highlights

- Three-dimensional ordered mesoporous carbon spheres modified with ultrafine zinc oxide nanoparticles are successfully prepared.
- The microwave absorbing performance of zinc oxide/carbon nanocomposites can be controlled through regulating ratio of zinc oxide nanoparticles.
- Electromagnetic simulation of radar cross section on a complicated groove structure demonstrates the microwave absorbing ability of the carbon based nanocomposites.

**Electronic supplementary material:**

The online version of this article (10.1007/s40820-021-00601-x) contains supplementary material, which is available to authorized users.

## Graphical abstract

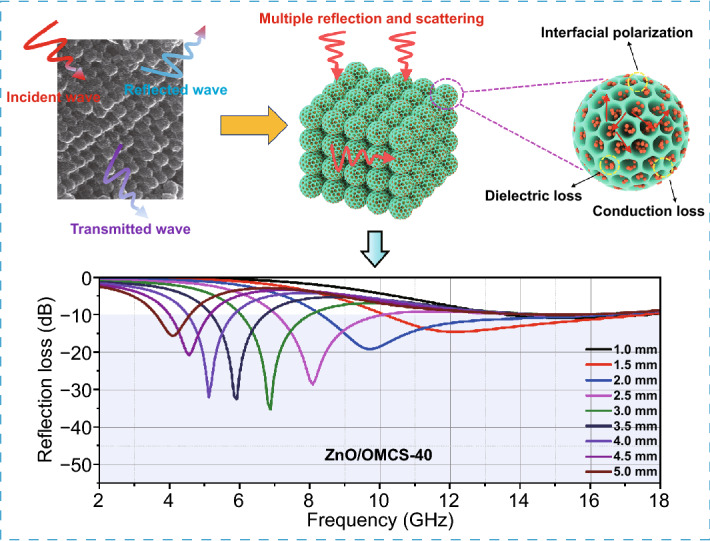

## Introduction

Since electromagnetic pollution became serious problem with the explosive development of electronics and wireless communication, the microwave absorption materials have gained great attention because of their ability to absorb and shield electromagnetic radiation [1–4]. At present, microwave absorbing materials feathered with strong absorption, small thickness, wide efficient absorption bandwidth and light weight have been considered as the idea candidate for attenuating electromagnetic energies [5–9].

Carbon materials are representative dielectric loss medium [10–14]. Nanostructured carbon materials, such as carbon black, carbon fibers, carbon nanotubes (CNTs), graphene and porous carbon, have attracted great interests as microwave absorbing materials for their low density and special physical and chemical properties [15–19]. Among them, the porous carbon involved in large pore volume, high-specific surface area and light weight gained much attention. Recent explorations have demonstrated that abundant porous configuration could benefit to the microwave absorbing performance [20–23]. The porous carbon materials, being a mixture of solid and air, could reduce the effective permittivity of carbon, while improve the impedance matching of materials, leading more electromagnetic waves into the structure [24–26]. Meanwhile, the pore structure, especially with three-dimensional porous structure, could extend the microwave transmission path of incident electromagnetic waves, resulting in multi-reflection and scattering in the porous materials [27]. The repeatedly multi-reflection and scattering offers more chance for the media to attenuate electromagnetic energies [28, 29]. Many porous carbon coupled with magnetic fillers such as magnetic ferrite, magnetic alloy, and metal–organic frameworks (MOFs) have been investigated [30–32]. For instance, Liu et al. developed a kind of MOF-derived carbon based nanocomposites. The magnetic nanocomposites reveal minimum RL (RL_min_) value of − 46.5 dB at 3.5 mm [33]. Yan et al. fabricated three-dimensional N-doped porous carbon foams embedded with CoNi alloy particles (CoNi@PRM-NC) as microwave absorbing materials. The 3D CoNi@PRM-NC achieves a RL_min_ value of − 56 dB at 17.8 GHz while its thickness is only 1.7 mm [34]. Although amazing microwave absorbing performance has been demonstrated, the large density of magnetic fillers has severely limited their practical applications.

Zinc oxide (ZnO), as a significantly lightweight, favorable dielectric and semi-conductive medium, has been extensively explored as microwave absorbing materials [35–38]. Meanwhile, to large-scale synthesize ZnO is easily realized and the low cost of preparing process is suitable for commercial application [39–41]. Up to now, some studies focusing on the microwave absorbing performance of porous carbon modified with ZnO have been reported [42]. For example, Wang et al. constructed a kind of hierarchical Ni/ZnO array hybrid nanostructures. The nanocomposites obtained a RL_min_ value of − 27.8 dB at 9.57 GHz with a wide effective absorption bandwidth of 4.2 GHz over 8–12 GHz [35]. Song et al. prepared three-dimensional reduced graphene oxide foams modified with ZnO nanowires. The RL_min_ value of the hybrids can be − 35.1 dB at 8.3 GHz [43]. These favorable microwave absorbing properties of ZnO nanocomposites can be mainly attributed to the increase of the effective polarization interfaces [44]. Interfacial polarization is significantly important to microwave attenuation. Interfacial polarization can be improved by enhancing the interface between different dielectric in microwave absorbing materials. The uniform distribution of ZnO are good for building up the interface of heterostructure. Meanwhile, the microwave impedance matching of porous carbon would be further improved by doping ZnO, which could reduce the reflection microwaves. However, uneven dispensability of ZnO in porous carbon has been a vexed problem, which may hinder the further improvement of microwave absorbing ability.

Herein, three-dimensional ordered mesoporous carbon spheres modified with ultrafine ZnO NPs (ZnO/OMCS) was rationally developed as high-performance microwave absorbing materials. The ultrafine ZnO NPs are uniformly distributed on the surface of three-dimensional ordered mesoporous carbon spheres, which is beneficial to the interfacial polarization and impendence match. Meanwhile, the 3D ordered mesoporous carbon spheres could promote the scattering and multiple reflection of incident microwaves. Thus, the ZnO/OMCS nanocomposites exhibits excellent microwave absorption ability and broad effective absorption bandwidth. The microwave attenuation mechanism for ZnO/OMCS nanocomposites was also investigated based on polarization loss, complex permittivity, and conductive loss. Furthermore, simulated radar cross section (RCS) results further demonstrated the ZnO/OMCS nanocomposites outstanding microwave absorbing ability on complex groove structure. This work indicates ZnO/OMCS nanocomposites as a promising microwave absorbing candidate material via low-cost and simple industrial processing.

## Experimental

### Materials

Methyl methacrylate (MMA, > 99.5%) was obtained from Aladdin. Tetraethoxysilane (TEOS, > 98%) was purchased from Tianjin Kemiou Chemical Reagent Co., Ltd, which need to further purify by vacuum distillation method. CH_3_OH (≥ 99.8%), CH_3_CH_2_OH (≥ 99.8%), Zn(CH_3_COO)_2_·2H_2_O (≥ 99%), KOH (≥ 85%), HCl (37%), and HF (≥ 40%) were acquired from Tianjin Kemiou Chemical Reagent Co., Ltd Triblock copolymer F127 (EO_106_PO_70_EO_106_) was obtained from Sigma-Aldrich Chemical Company. Resol (Mw < 500) was prepared by published strategy [45].

### Synthesis of ZnO/OMCS

**Scheme 1:**
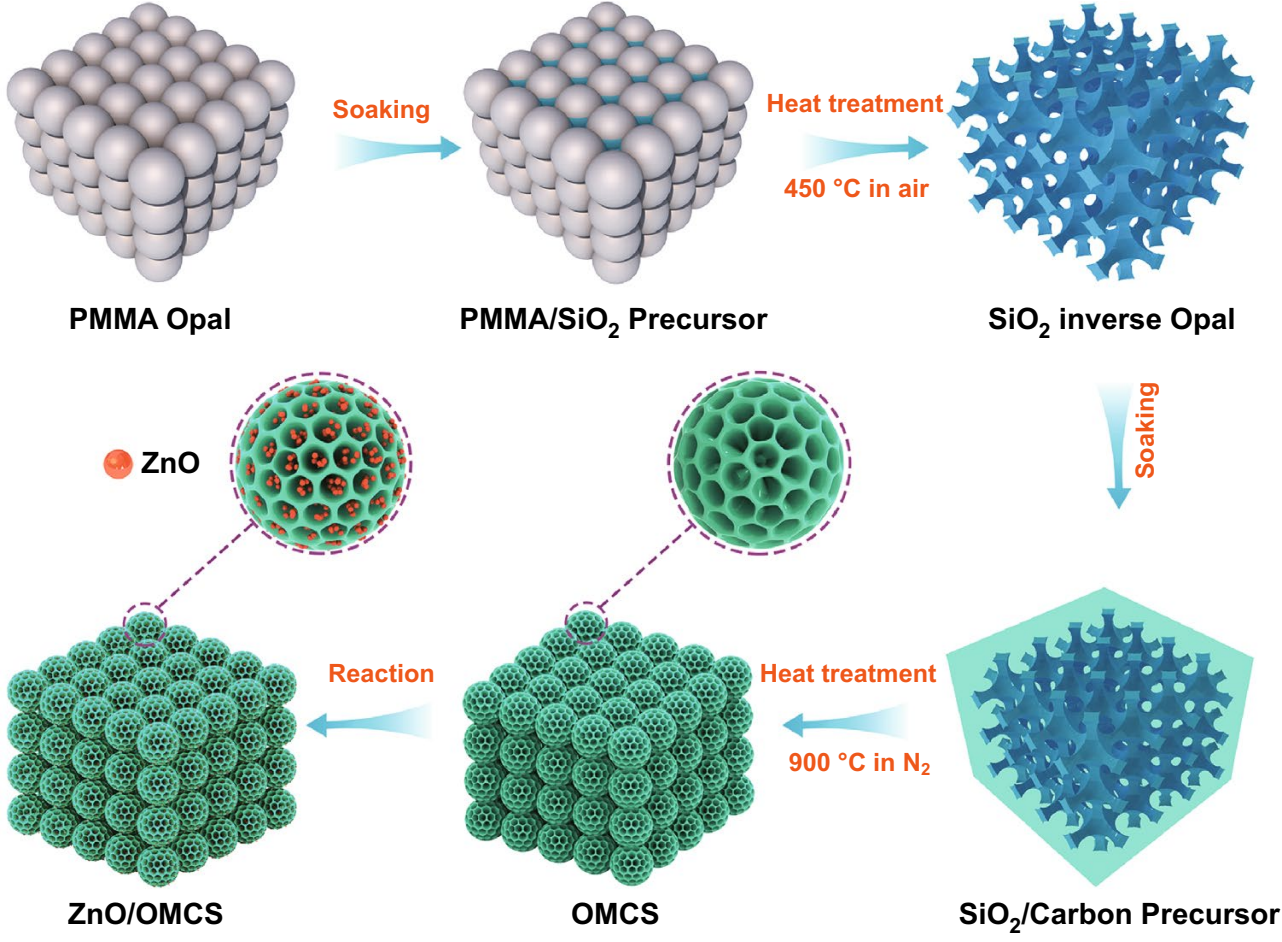
Schematic illustration on the synthesis of ZnO/OMCS

Scheme 1 shows the synthesis process of ZnO/OMCS and the details are as follows. The poly (methyl methacrylate) (PMMA) colloidal crystal used as hard template to fabricate silica inverse opal is firstly fabricated according to the reported method [46]. Silica precursor was prepared by mixing TEOS, 0.1 M HCl and ethanol with a mass ratio of 1: 1: 1.5 under magnetic stirring for 1 h. A piece of PMMA template was soaked in above silica precursor solution and stand still for 1 h. The impregnated PMMA template was then removed from the solution and dried at room temperature, followed by heated at 450 °C in an oven for 5 h to remove PMMA template. The obtained silica inverse opal was then applied as second step template. A few pieces of silica template were impregnated in 20 mL of ethanol solution containing 1 g of resol and 1 g of F127. After the ethanol solution was evaporated completely at room temperature, the precursor/silica composite was heated at 100 °C for 24 h. Under nitrogen atmosphere, the composite is heated at 350 °C for 5 h to remove F127 at a heating rate of 1 °C min^−1^. Subsequently, the temperature was raised to 900 °C at a heating rate of 5 °C min^−1^ and kept for 2 h for carbonization of resol. Next, the prepared carbon/silica composite was immersed in HF solution (5%) for 3 days to etch the silica template. The produced 3D ordered mesoporous carbon spheres (OMCS) was then used as support to host ZnO NPs by sol–gel method as follows. First, 0.5 g of OMCS was ultrasonically dispersed in 25 mL methanol solution containing 0.9 g of Zn(CH_3_COO)_2_•2H_2_O for 1 h. Then, 20 mL of 0.45 M KOH methanol solution was slowly added in above methanol solution under magnetic stirring. After 1 h of reaction, the black powder (Zn(OH)_2_/OMCS) was collected by filtration. Finally, ZnO/OMCS was produced by heating the black powder at 70 °C overnight. The theoretical ZnO content in ZnO/OMCS composite is ~ 40 wt.%. For comparison, the composites with lower ZnO loadings (denoted as ZnO/OMCS-20 and ZnO/OMCS-30, the theoretical ZnO content is 20 wt.% and 30 wt.%) were prepared by the same steps but the addition of Zn source is 0.34 g and 0.58 g, respectively.

### Material characterization

The morphology and structure of samples were characterized by scanning electron microscopy (SEM, JEOL JSM-7100F) and transmission electron microscope (TEM, JEOL JEM-2010, 200 kV). Powder X-ray diffraction (XRD) data of ZnO/OMCS was obtained from Rigaku D/MAX 2200 VPC equipment with Cu Kα radiation. The Brunauer–Emmett–Teller (BET) data was acquired on Micro-meritics ASAP-2020 instrument. The actual content of ZnO in composite was obtained by SDT Q-600 equipment. The X-ray photoelectron spectra (XPS) were carried out on PHI 5000 Versa Probe system.

### Measurements

The vector network analyzer (Keysight Technologies, N5244B) was employed to measure the basic electromagnetic parameters of all samples. ZnO/OMCS nanocomposites were mixed with pure paraffin at the ratio of 3:7. The mixture was pressed into a circular ring shape of out diameter of 7.0 mm, inner diameter and height of 3.0 mm. The RL values were carefully calculated according to the measured electromagnetic parameters. RCS values of the multi groove structure were simulated according to the measured electromagnetic parameters.

### Calculation details

The adsorption behavior and electronic structures of graphite/ZnO are performed using software of CASTEP, which is constructed based on density functional theory (DFT) [47]. In the calculation process, method of ultrasoft pseudopotentials was used. Exchange–correlation functional of generalized gradient approximation (GGA) in Perdew-Burke-Ernzerhof (PBE) was employed. The valence configuration of the calculated atoms was: 2s^2^2p^2^ for C, 2s^2^2p^4^ for O, and 3d^10^4s^2^ for Zn. Cutoff energy for the plane-wave basis was 350 eV. The k-point meshing was set to be 6 × 6 × 1 to sample the Brillouin zone. The valence configuration of the calculated atoms were: 2s^2^2p^2^ for C, 2s^2^2p^4^ for O, and 3d^10^4s^2^ for Zn. Firstly, graphite (0001) surface slab with three atomic layers was built, and a 16 Å-thick vacuum layer was added. To avoid the interaction between neighbor ZnO molecules, a 2 × 2 × 1 supercell of graphite was constructed. Then a molecule of ZnO was placed right above the graphite. The model experienced geometry optimization. During the relaxation process, the positions of C atoms were fixed and molecule of ZnO could relax freely. The convergence tolerances of energy, maximum displacement, maximum force, and maximum stress are 1.0 × 10^–6^ eV atom^−1^, 1.0 × 10^–3^ Å, 0.03 eV Å^−1^, and 0.05 GPa, respectively.

## Results and discussion

### Preparation and characterization of ZnO/OMCS nanocomposites

**Fig. 1:**
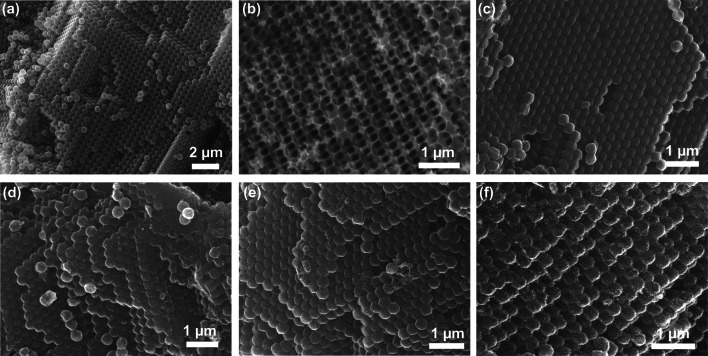
SEM images of **a** PMMA opal, **b** silica inverse opal, **c** OMCS, **d** ZnO/OMCS-20, **e** ZnO/OMCS-30 and **f** ZnO/OMCS-40

Figure 1a depicts the typical SEM image of PMMA colloidal crystal composed of 3D ordered PMMA spheres (460 nm in diameter). Figure 1b is an SEM image of silica inverse opal fabricated from PMMA template, showing 3D ordered interconnected macropores with average pore size of 410 nm. The silica inverse opal is applied as second template to prepare OMCS, which is shown in Fig. 1c. As can be observed, OMCS demonstrates 3D ordered spherical array structure and the average diameter of the carbon spheres is ca. 380 nm. Because of the condensation of silica and carbon precursor during heating process, the mean size of carbon spheres is smaller than that of the original PMMA spheres after two-step reverse replication procedure. Figure 1d–f displays the SEM images of ZnO/OMCS-20, ZnO/OMCS-30, and ZnO/OMCS-40 prepared by loading ZnO NPs on OMCS. It can be found that the closed packed structure of carbon spheres is well preserved, indicating that the loading process of ZnO would not destroy the 3D ordered structure of OMCS.

**Fig. 2:**
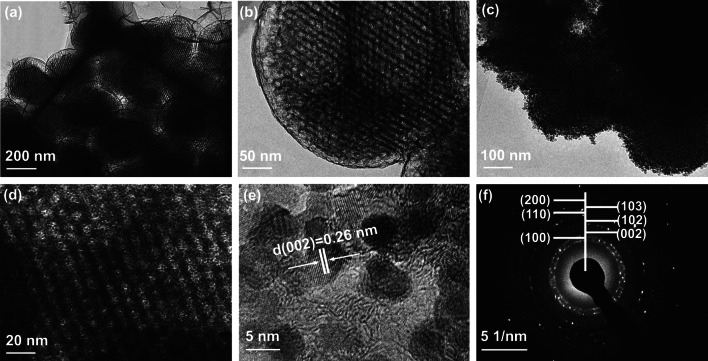
**a, b** TEM images of OMCS with different magnifications. **c, d** TEM images of ZnO/OMCS-40 with different magnifications. **e** HR-TEM image of ZnO/OMCS-40. **f** The corresponding SAED pattern of ZnO/OMCS-40

TEM was employed to characterize the porous structure of the prepared samples. The low magnification TEM image of OMCS (Fig. 2a) illustrate that ordered mesoporous structure exist in each carbon spheres. The average mesopore size is ~ 12 nm calculated from the high magnification TEM image (Fig. 2b). These relatively large mesopores can facilitate the growth of ZnO NPs on mesoporous walls of OMCS. Figure 2c shows the TEM image of ZnO/OMCS-40 and there are no bulk ZnO can be seen, implying that ZnO NPs are uniformly distributed on carbon spheres. The higher-magnification view of ZnO/OMCS-40 (Fig. 2d) reveals that the mesoporous structure still maintained after deposition of ZnO NPs. There preserved mesoporous structure is in favor of the microwave absorbing ability. As can be seen from HR-TEM image of ZnO/OMCS-40 (Fig. 2e), the ZnO NPs are uniform in the mean diameter of ~ 5 nm, and exhibit a polycrystalline wurtzite structure with lattice fringe of 0.26 nm that assigned to ZnO (002) plane [37]. The selected area electron diffraction (SAED) pattern with bright and continuous diffraction rings in Fig. 2f also confirms the polycrystalline behavior of ZnO. Figure S1 depicts the TEM images of ZnO/OMCS-20 and ZnO/OMCS-30 composites and their corresponding SAED patterns. As can be observed from Fig. S1a, c, after loading of ZnO, some mesopores in carbon spheres are still observed. From SAED patterns (Figs. S1b, d and 1f), the diffraction rings composed of dots are getting brighter with the increase of ZnO content, suggesting more polycrystalline ZnO on carbon spheres.

**Fig. 3:**
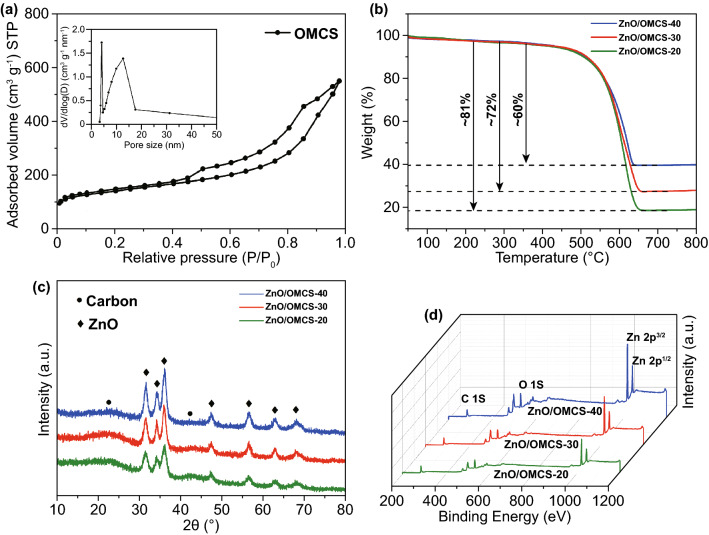
**a** N_2_ adsorption–desorption isotherms and pore size distribution of the OMCS. **b** TGA profiles, **c** XRD patterns, and **d** XPS spectra for the ZnO/OMCS-20, ZnO/OMCS-30 and ZnO/OMCS-40 nanocomposites

The porous structure of OMCS can further be estimated by nitrogen adsorption–desorption isotherms. As shown in Fig. 3a, type-IV isotherm and a hysteresis loop of type H1 can be observed, indicating that OMCS possesses mesoporous structure [48]. The BET surface area and pore volume of OMCS are 537.3 m^2^ g^−1^ and 0.72 cm^3^ g^−1^. According to the pore size distribution (PSD) curve derived from nitrogen gas adsorption, the mesopore sizes of OMCS are mainly distributed in the diameter range of 8–15 nm, centered at 12.4 nm. This result is consistent with TEM observation (Fig. 2b). These mesopores can restrict the growth and agglomeration of ZnO NPs. After loading ZnO nanoparticles on OMCS, the mesoporous structure still exists, which can be proved by the H1 type hysteresis loops in the isotherms of the ZnO/OMCS composites (Fig. S2). ZnO/OMCS-20 shows a peak located at 5.6 nm in the PSD curve (inset of Fig. S2), smaller than that of the OMCS, implying that the ZnO NPs are deposited on the mesopore walls of the OMCS. These is no peak in the range of 5–15 nm in the PSD curves for the ZnO/OMCS-30 and ZnO/OMCS-40 composites, which is due to the large amount of ZnO NPs that located in the mesopores, destroying the uniformity of mesopores. In addition, the specific surface area and pore volume of the composite decrease with the increase in ZnO content (Table S1), also confirming that more and more ZnO NPs are deposited in the mesopores of OMCS.

The actual loading of ZnO on the OMCS can be evaluated by TGA method and the TGA curves of nanocomposites are shown in Fig. 3b. Each TGA curve shows one weight loss appeared between 450 and 600 °C, corresponding to the oxidation of carbon. The actual contents of ZnO in the nanocomposites are calculated to be ~ 19%, ~ 28%, and ~ 40% for ZnO/OMCS-20, ZnO/OMCS-30, and ZnO/OMCS-40. This result agrees with the theoretical value, suggesting that the ZnO content in the nanocomposite can be precisely controlled by the adding amount of Zn sources.

XRD is applied to determine the crystal structure of composite, and the XRD patterns for ZnO/OMCS composites are shown in Fig. 3c. All XRD patterns show two slight bulges at 2θ value of 23° and 43°, assigning to the (002) and (100) reflection of graphitic planes [49]. The other diffraction peaks in the XRD curves correspond to the hexagonal wurtzite structure of ZnO (JCPDS No. 36-1451) [50]. XRD results also verify that the ZnO nanoparticles are successfully deposited on carbon spheres. Additionally, XRD can be used to determine the ZnO crystallite size by applying Scherrer equation from the full width at half maximum (FWHM) of the diffraction peak at 56.5°. After calculating, the crystallite sizes of ZnO in the composites are 3.6, 4.7, and 5.5 nm for ZnO/OMCS-20, ZnO/OMCS-30, and ZnO/OMCS-40, respectively. These ultrafine ZnO NPs are benefit to the multiple reflection and scattering of incident microwave.

The presence of C, O, and Zn surface species also can be confirmed by the full survey XPS spectra of ZnO/OMCS composites, as shown in Fig. 3d. And there are no other species can be detected, indicating the purity of the composites. In addition, the character peaks of O and Zn species are getting stronger during the ZnO content increasing, verifying the ZnO nanoparticles has been successfully deposited on the surface of OMCS.

**Fig. 4:**
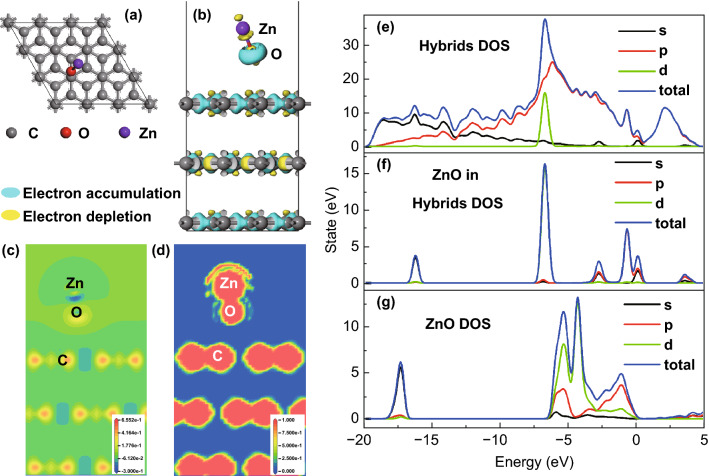
Simulation results of ZnO/OMCS. **a** Top view of graphite/ZnO structure after relaxation; **b** side view of charge density difference map with isovalue of 0.1; slice of **c** charge density difference and **d** electron localization functional along (011) plane. DOS results for **e** graphite/ZnO hybrids, **f** ZnO in hybrids, **g** bare ZnO molecule

The structure of ZnO/OMCS was studied by first-principles calculations to further confirm the experimental results. The calculated electron density difference map is shown in Fig. 4. Figure 4a shows the top view of graphite/ZnO structure after relaxation. The binding behavior of ZnO molecule on the graphite (0001) surface is studied with adsorption energy (*E*_ad_). *E*_ad_ can be obtained by Eq. 1 [51]:

1 $$E_{{{\text{ad}}}} = E_{{{\text{graphite}}/{\text{ZnO}}}} - E_{{{\text{graphite}}}} - E_{{{\text{ZnO}}}}$$

where, *E*_graphite/ZnO_ is the energy of graphite/ZnO structure after relaxation, the *E*_graphite_ and *E*_ZnO_ are the energies of bare graphite (0001) slab and ZnO molecule. The adsorption height (h) was also measured. Value of h can be defined as the vertical distance between O and the topmost C layer.

The calculated values of *E*_ad_ and *h* are − 3.65 eV and 2.79 Å. Figure 4b shows the charge density difference map with isovalue of 0.1. In the map, the light blue color represents charge accumulation area and the yellow color represents charge depletion area. Figure 4c shows slice of charge density difference along (011) plane. From the results of charge density difference, it is found that some valance electrons are transferred from Zn atom to the O atom. As a result, Zn and O atoms become anions and cations and bonds with each other through electrostatic attraction. Therefore, Zn–O bond is typical ionic bond. We also found that ZnO has influence on the charge distribution on the surface of graphite. A small amount of electrons is transferred from C atom to the O atom. Figure 4d shows slice of electron localization functional (ELF) along (011) plane. The data inside the core region are meaningless for the pseudopotentials were used in the calculation process. There are some localized electrons between Zn and O atoms, indicating that Zn–O bond also has some composition of covalent bond. As to C=C bond in the graphite, large amount electrons are found between neighbor C atoms, which is typical feature of π-π covalent bond in hexagonal (0001) plane.

The result of density of states (DOS) and partial density of states (PDOS) can provide more information about the nature of interaction between ZnO and graphite. Figure 4e–g shows the DOS results of graphite/ZnO hybrids, ZnO hybrids and bare ZnO molecule, respectively. Comparing the DOS of bare ZnO, positions of some peaks for ZnO in graphite/ZnO hybrids are changed. What is more, some new peaks appeared, which corresponds to the peaks of graphite. The overlapping confirms the hybridization of O 2sp, Zn sd, and C 2sp orbitals.

### Microwave absorption performance of the ZnO/OMCS nanocomposites

**Fig. 5:**
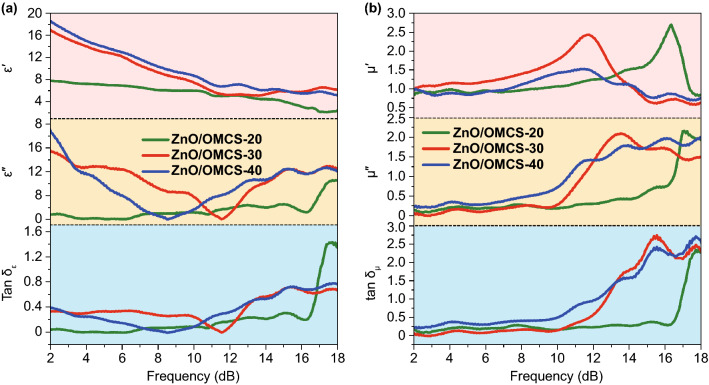
Frequency dependence of **a** real part of permittivity, imaginary part of permittivity and tangent dielectric loss values of ZnO/OMCS-20, ZnO/OMCS-30 and ZnO/OMCS-40 **b** real parts of permeability, imaginary part of permeability and tangent magnetic loss values of ZnO/OMCS-20, ZnO/OMCS-30 and ZnO/OMCS-40

The electromagnetic parameters of ZnO/OMCS nanocomposites are plotted in Fig. 5 to investigate the effect of composition and microstructure on microwave absorbing performance. The real part (*ε*') and imaginary part (*ε*″) of dielectric constant values of the ZnO/OMCS-20 vary from 7.9 to 2.1 and 0 to 3.2, respectively. After introducing more ultrafine ZnO NPs into OMCS, ε' and ε″values of ZnO/OMCS-30 and ZnO/OMCS-40 in most of the frequency range reveal a significant increase, indicating the strong dielectric loss ability. According to the previous calculation results of first-principles, the electron density is different between ZnO and carbon base, which could result in interfacial polarization. The increase in ZnO NPs distributed on the OMCS would not only enhance the associated interfacial polarization, but also build up the active interfaces on the base of 3D ordered porous structure. It is known that the higher tan *δ*_*ε*_ value means more dissipation of electromagnetic energy. The tan *δ*_*ε*_ values of ZnO/OMCS-30 and ZnO/OMCS-40 are heavily intertwined, while their tan *δ*_*ε*_ values are larger than that of ZnO/OMCS-20 in most of the frequency range. This provides the clear evidence that the uniformly distributed ultrafine ZnO NPs are helpful to enhance the dielectric loss ability. Meanwhile, resonant peaks were observed in the *ε″* curves of ZnO/OMCS nanocomposites. The resonant peaks can be ascribed to the interfacial polarization and dipole polarization at 2–18 GHz. According to Debye relaxation theory, the relation between ε′ and ε′′ follows Eq. 2 [52].

2 $$\left( {\varepsilon^{\prime } - \frac{{\varepsilon_{s} + \varepsilon_{\infty } }}{2}} \right)^{2} + (\varepsilon^{\prime \prime } )^{2} = \left( {\frac{{\varepsilon_{s} - \varepsilon_{\infty } }}{2}} \right)^{2}$$

Generally, a single semicircle derived from the plot of *ε*′ versus *ε*′′ was regarded as the Cole–Cole semicircle. Every semicircle represents one Debye relaxation process. Once a polarization relaxation process happens, Eq. 2 can be used to describe the relationship between *ε*′ and *ε*″.

**Fig. 6:**
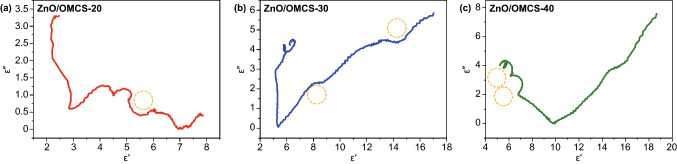
Typical Cole–Cole semicircles (*ε*′ vs. *ε*′′) for **a** ZnO/OMCS-20, **b** ZnO/OMCS-30, **c** ZnO/OMCS-40

The plots of *ε*′–*ε*′′ curves of ZnO/OMCS nanocomposites were shown in Fig. 6. Obviously, the plots of ZnO/OMCS-20 in Fig. 6a reveal less distorted Cole–Cole semicircles than that of ZnO/OMCS-30 and ZnO/OMCS-40. This indicates a stronger polarization relaxation occurs in ZnO/OMCS-30 and ZnO/OMCS-40, which generated at the interfaces between ZnO NPs and 3D porous carbon base. The dependences of *μ*′ and *μ*′′ at 2–18 GHz of ZnO/OMCS nanocomposites are shown in Fig. 5b. The μ′ values of ZnO/OMCS are almost the same in the whole frequency. However, ZnO/OMCS-30 and ZnO/OMCS-40 possess obviously larger *μ*′′ values than that of ZnO/OMCS-20 since 10.2 GHz. This indicates that ZnO/OMCS-30 and ZnO/OMCS-40 exhibit the optimized magnetic loss performance. Magnetic loss tangent values show the same trend of *μ*′′ values.

**Fig. 7:**
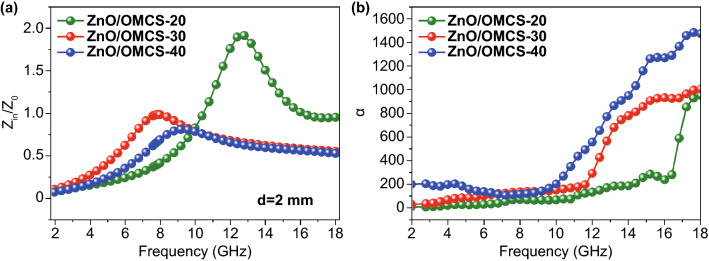
**a** Relative input impedance of ZnO/OMCS-20, ZnO/OMCS-30, ZnO/OMCS-40 at 2 mm. **b** Attenuation constant of ZnO/OMCS-20, ZnO/OMCS-30, ZnO/OMCS-40

The modulus of the normalized characteristic impedance and attenuation constant (*α*) of ZnO/OMCS nanocomposites are further investigated. The modulus of the normalized characteristic impedance *Z* =|*Z*_in_/*Z*_0_| can be calculated according to Eq. 3, which represents the ability of the microwave entering into the absorbing materials. If *Z* is close to 1, it indicates the material possess a good impedance matching characteristic. At the thickness of 2 mm, ZnO/OMCS-20 represents the worst impedance matching characteristic while ZnO/OMCS-30 obtains the best impedance matching characteristic (Fig. 7a) [53].

3 $$Z = \left| {Z_{in} /Z_{0} } \right| = \sqrt {\left| {\mu_{r} /\varepsilon_{r} } \right|} \tanh \left[ {j\left( {\frac{2\pi fd}{c}} \right)} \right]\sqrt {\mu_{r} \varepsilon_{r} }$$

4 $$RL = 20\log \left| {(Z_{{{\text{in}}}} - Z_{0} )/(Z_{{{\text{in}}}} + Z_{0} )} \right|$$

Here, *Z*_in_ represents the normalized input impedance of the microwave absorbing materials, *Z*_0_ is the impedance of free space, *ε*_*r*_ and *μ*_*r*_ are the complex permittivity and complex permeability, respectively. Attenuation constant *α* can be expressed as Eq. (5) [54]:

5 $$\alpha = \frac{\sqrt 2 \pi f}{c} \times \sqrt {(\mu^{\prime \prime } \varepsilon^{\prime \prime } - \mu^{\prime } \varepsilon^{\prime } ) + \sqrt {\left( {\mu^{\prime \prime } \varepsilon^{^{\prime\prime}} - \mu^{\prime } \varepsilon^{\prime } } \right)^{2} + (\mu^{\prime } \varepsilon^{^{\prime\prime}} + \mu^{^{\prime\prime}} \varepsilon^{\prime } )^{2} } }$$

Attenuation constants of ZnO/OMCS nanocomposites are shown in Fig. 7b. The order of attenuation constants of three samples is ZnO/OMCS-40 > ZnO/OMCS-30 > ZnO/OMCS-20.

**Fig. 8:**
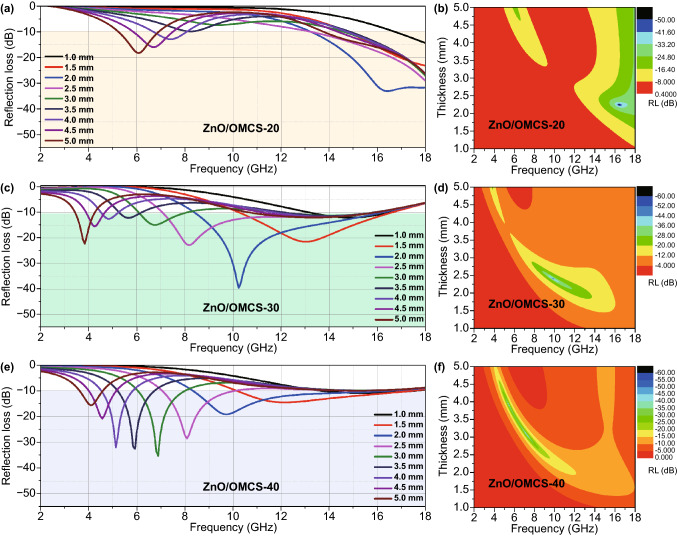
Reflection loss and Delta value maps of ZnO/OMCS with different ZnO contents. **a, b** ZnO/OMCS-20; **c, d** ZnO/OMCS-30; **e, f** ZnO/OMCS-40

**Fig. 9:**
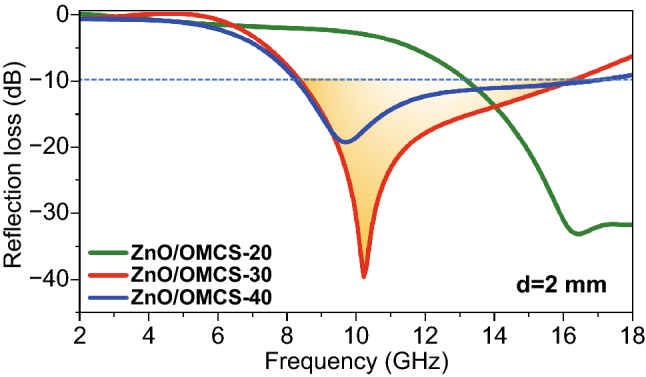
Reflection loss for ZnO/OMCS with different ZnO contents under a constant thickness of 2 mm

The reflection loss of ZnO/OMCS nanocomposites are calculated according to Eq. 4. The corresponding reflection loss color maps of ZnO/OMCS-20, ZnO/OMCS-30 and ZnO/OMCS-40 as a function of frequency and thicknesses are shown in Fig. 8. Obviously, ZnO/OMCS-20 exhibits relative poor microwave absorbing performance. Comparatively, ZnO/OMCS-30 exhibits an effective absorption bandwidth of 6.8 GHz (10.4 to 17.2 GHz) at 1.5 mm, 8.5 GHz (8.3 to 16.8 GHz) at 2.0 mm and 9.3 GHz (7.1 to 16.4 GHz) at 2.5 mm (Fig. 8c, d). The ZnO/OMCS-30 holds an RL_min_ of -39.3 dB at the frequency of 10.4 GHz with a small thickness of 2.0 mm, indicating the strong absorption ability [4]. Meanwhile, ZnO/OMCS-40 also shows a wide absorption bandwidth. It reveals an effective absorption bandwidth of 7.6 GHz (10.1 to 17.7 GHz) at 1.5 mm and 9.1 GHz (8.2 to 17.3 GHz) at 2.0 mm. The reflection loss values of three samples at 2.0 mm are shown in Fig. 9, which means the microwave absorption performance of ZnO/OMCS nanocomposites can be tuned by changing the ZnO NPs content. The significant feather of ZnO/OMCS nanocomposites such as lightweight, strong absorption and wide band width shows it great potential as excellent microwave absorbing materials [14].

**Fig. 10:**
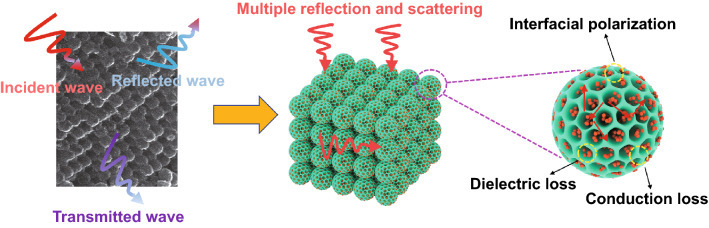
Schematic illustration of the microwave absorbing mechanism of ZnO/OMCS

The supposed microwave absorbing mechanism of ZnO/OMCS nanocomposites is as follows (Fig. 10). Firstly, the three-dimensional ordered mesoporous carbon spheres of the carbon base provide abundant solid-air interfaces to realize the impendence matching condition. Second, conduction loss, multiple reflection, and scattering loss of microwaves can be promoted by the 3D ordered porous structure. Third, the simulation results have demonstrated that the uniform distribution of ultrafine ZnO NPs is benefit to the polarization interface and the defect-dipoles induced by oxygen vacancies in the ZnO crystal promotes polarization loss. Generally, it is known that nanoparticles are easily to be self-assembled due to their high surface energy. This aggregation problem can be effectively solved by the three-dimensional ordered mesoporous carbon spheres structure. The mesoporous carbon spheres limited the growth of ZnO NPs, which makes them ultrafine size. Therefore, the ZnO NPs are effectively separated and distributed in the porous structure rather than compactly aggregated, which benefits the formation of denser dielectric coupling network and the enhancement of dielectric loss ability. Meanwhile, the three-dimensional ordered mesoporous carbon spheres structure can increase the loading ratio of ZnO NPs. More ZnO NPs can increase the number of active sites in the nanocomposites, which is good for scattering loss.

### Radar cross section

The rough multiple groove structure is commonly used in ground armor and is a strong electromagnetic scattering source. In order to evaluate the electromagnetic energy dissipation ability of ZnO/OMCS, the simulation of RCS values of a metal plate with multiple groove structures was selected to demonstrate the microwave absorption performance of ZnO/OMCS.

**Fig. 11:**
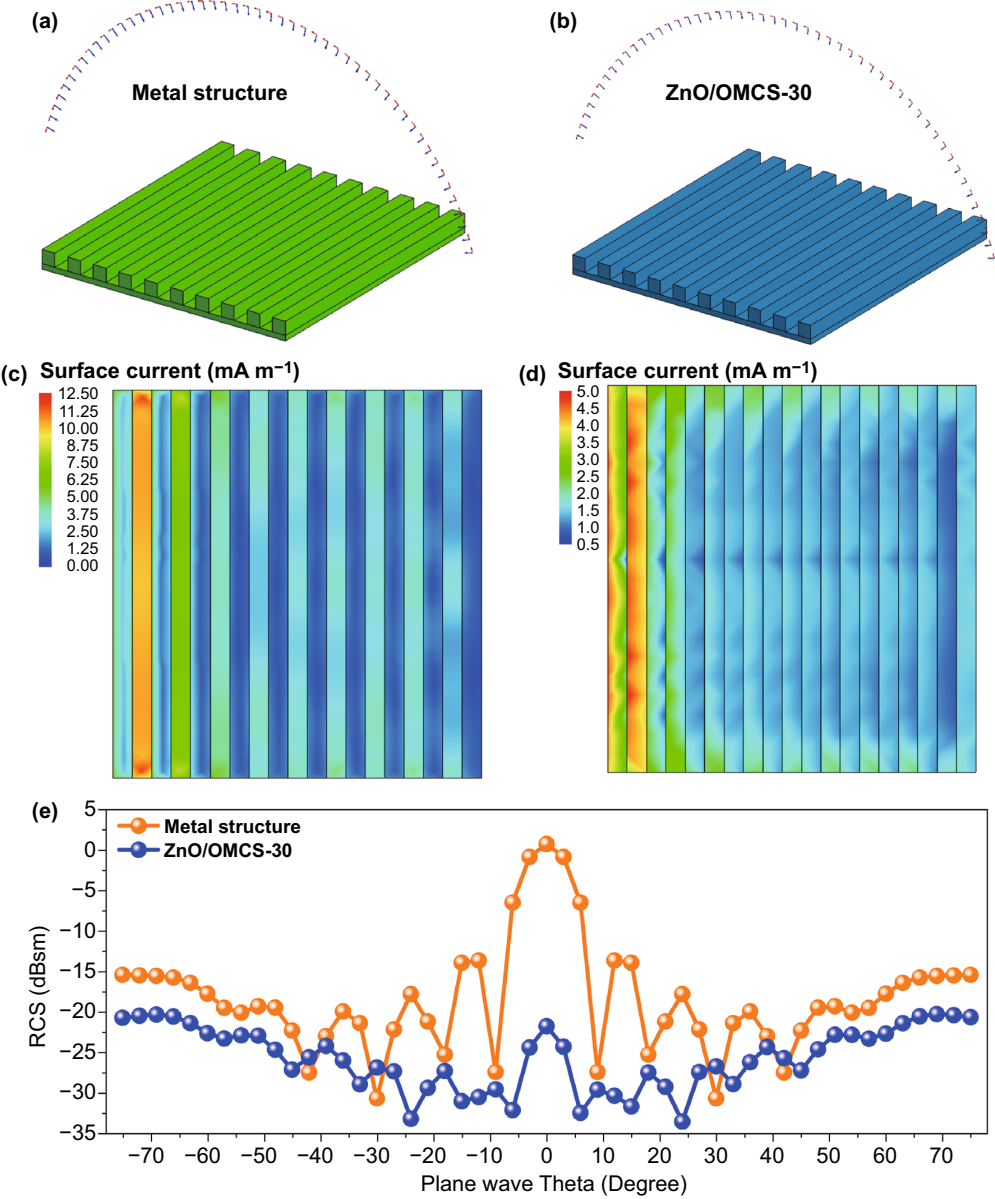
RCS simulation results of a rectangle metal plate and a rectangle metal plate with ZnO/OMCS. **a, b** The model of metal plate and metal plate with ZnO/OMCS-30 coatings. **c, d** Metal plate models and corresponding color maps of simulated current distributions. **e** RCS values of metal structure and metal structure with ZnO/OMCS coatings

X-band radar is widely used and significantly important in national defense [55–57]. Thus, the simulation frequency was chosen at 10.4 GHz. The RCS characteristics of the metal groove structures (Fig. 11a) and metal groove structures with ZnO/OMCS-30 coatings (Fig. 11b), thickness of coating is 2 mm) from − 90° to + 90° are simulated by method of moment. The simulation procedures are shown in the Supporting Information. The simulated surface current distribution on groove structure are shown in Fig. 11c, d. Clearly, the ZnO/OMCS-30 coatings effectively suppress the electromagnetic wave scattering on the metal groove structure. The corresponding RCS simulation results are shown in Fig. 11e. It can be observed that the RCS values of metal groove structure with ZnO/OMCS-30 coatings are much smaller than that of metal groove structure. Generally, the strongest electromagnetic scattering appears at zero degree for plate structure. However, it can be observed that the RCS value at this zero degree was decreased from 1.7 to − 20.6 dBsm after introducing ZnO/OMCS-30 coatings. These results demonstrated the ZnO/OMCS nanocomposites reveal good microwave absorbing ability and could effectively suppress the strong electromagnetic scattering of metal groove structure.

## Conclusions

3D ordered ZnO/OMCS nanocomposites were prepared as high-performance microwave absorbing materials. ZnO/OMCS-30 nanocomposites show effective absorption bandwidth of 9.1 GHz at 2.0 mm from 8.3 to 16.8 GHz and a strong absorption of − 39.3 dB at the frequency of 10.4 GHz with a thickness of 2 mm. ZnO/OMCS-40 nanocomposites also exhibit favorable microwave absorbing performance in the lower frequency. RCS simulation demonstrated the ZnO/OMCS nanocomposites remarkable properties in suppressing strong electromagnetic wave scattering of metal groove structure. The ZnO/OMCS nanocomposites with light weight, strong absorption, and wide band width yield various insights into the development of stealth technologies.

## Supplementary Information

Supplementary file1 (DOCX 667 kb)
