# Supplementary material for: Three-Dimensional Ordered Mesoporous Carbon Spheres Modified with Ultrafine Zinc Oxide Nanoparticles for Enhanced Microwave Absorption Properties
Source: Nanomicro Lett. 2021 Feb 17;13:76. doi: 10.1007/s40820-021-00601-x (PMC8187605; doi:10.1007/s40820-021-00601-x)
Supplement: Supplementary file 1 — Supplementary file1 (DOCX 667 kb) [file 40820_2021_601_MOESM1_ESM.docx]

Supporting Information for

**Three-****dimensional ordered mesoporous carbon spheres modified with ultrafine zinc oxide nanoparticles for enhanced microwave absorption properties**

Yan Song^1^, Fuxing Yin^1^, Chengwei Zhang^1,^ *, Weibing Guo^1,^ *, Liying Han^1^, Ye Yuan^1,^ *

^1^School of Materials Science & Engineering, Tianjin key laboratory of materials laminating fabrication and interface control technology, Hebei University of Technology, Tianjin 300130, People’s Republic of China

*Corresponding authors. E-mail: cwzhang@hebut.edu.cn (Chengwei Zhang);

gwbingo@163.com (Weibing Guo); yuanyewins@hotmail.com (Ye Yuan)


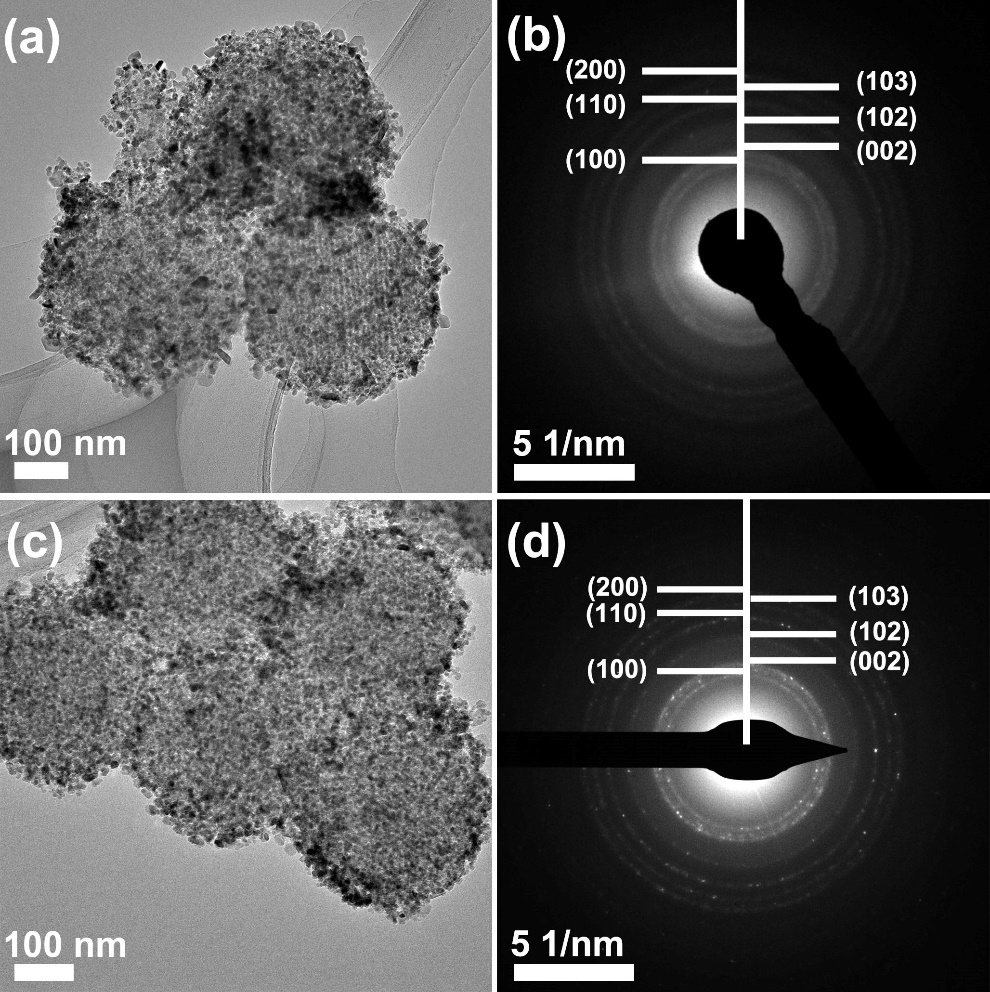


**Fig. S1. a** TEM image of ZnO/OMCS-20, **b** the SAED pattern of ZnO/OMCS-20_,_ **c** TEM image of ZnO/OMCS-30, and **d** SAED pattern of ZnO/OMCS-30.


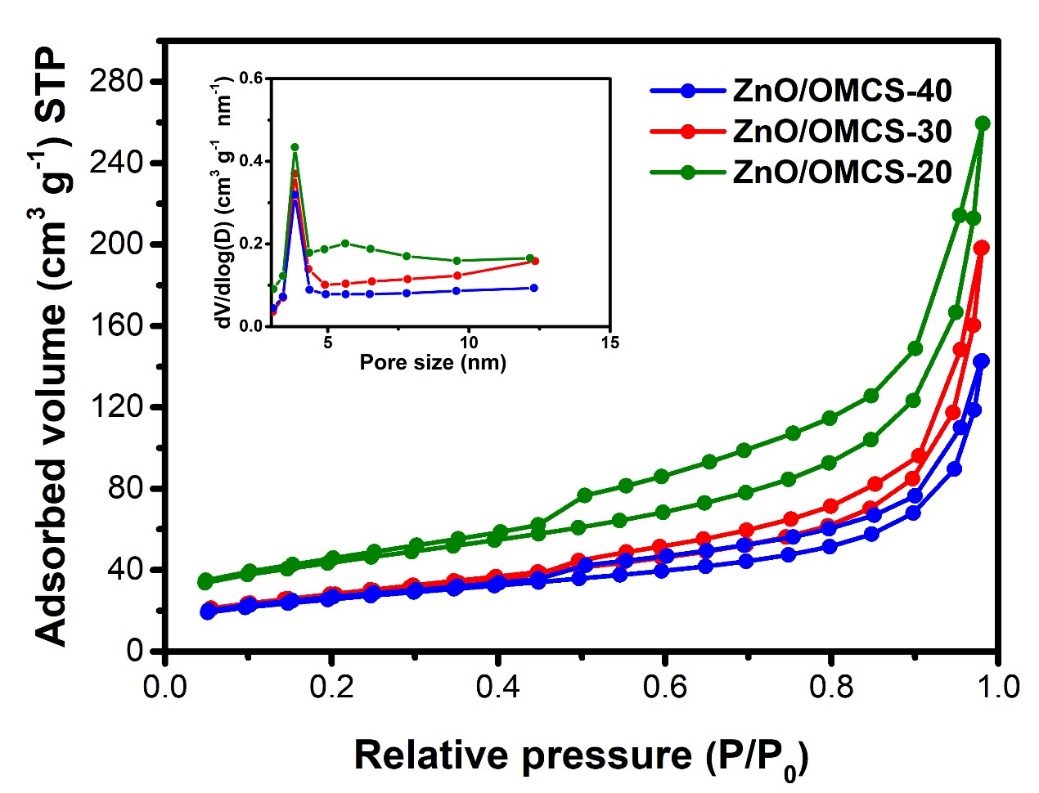


**Fig. S2.** N_2_ adsorption/desorption isotherms and pore size distributions for the ZnO/OMCS-20, ZnO/OMCS-30 and ZnO/OMCS-40 nanocomposites.

**Table S1 BET surface areas (*S*), pore volumes (*V*) and pore sizes (*D*) for the prepared samples.**

| samples | *S^a^* (m^2^ g^-1^) | *V*^b^ (cm^3^ g^-1^) | *D*^c^ (nm) |
| --- | --- | --- | --- |
| OMCS | 537.3 | 0.72 | 12.4 |
| ZnO/OMCS-20 | 155.9 | 0.37 | 5.6 |
| ZnO/OMCS-30 | 103.1 | 0.3 | — |
| ZnO/OMCS-40 | 92.4 | 0.2 | — |

^a^The Brunauer-Emmett-Teller (BET) surface area was measured by applying the linear part of the BET plot.

^b^The pore volume is calculated using adsorption isotherms by BJH method.

^c^The pore size is referred to the peak position in Fig. 3a and Fig. S2.

**Radar cross section (RCS) simulation**

**Geometry settings:** The width of the metal groove structure is 100 ×100 mm, the width of the groove is 10 mm while the height of the groove is 5 mm.

**Frequency:** The frequency of the incident waves is 10.4 GHz.

**Excitation source:** Plane waves were chosen as excitation source. The start θ is -90^o^ and the end θ is 90 ^o^.


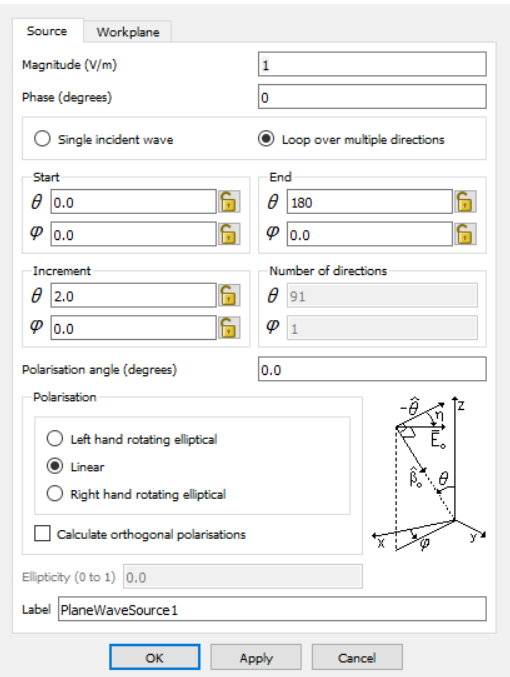


**Simulation method:** The moment of method was chosen to simulate the surface current distribution and radar cross section.
